# Supplementary figures and images for: A double-negative feedback loop between miR319c and JAW-TCPs establishes growth pattern in incipient leaf primordia in Arabidopsis thaliana
Source: PLoS Genet. 2023 Sep 28;19(9):e1010978. doi: 10.1371/journal.pgen.1010978 (PMC10564139; doi:10.1371/journal.pgen.1010978)

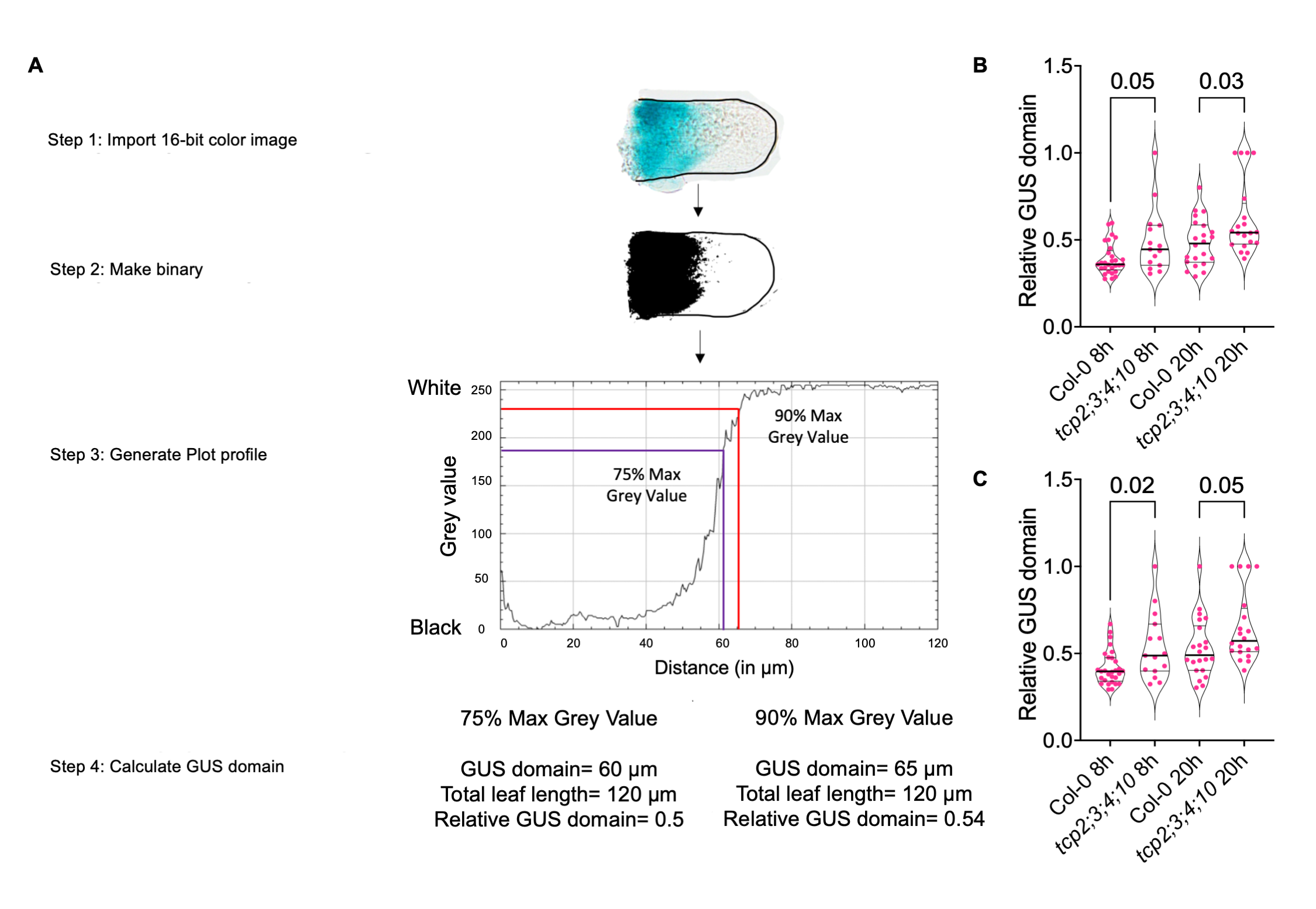

Supplement: S1 Fig — (A) Schematic representation of the pixel density-based method for quantification of GUS expression domain in leaf primordia. Domain of GUS activity was determined using the plot profile function of the ImageJ software (https://imagej.nih.gov/nih-image/more-docs/Tutorial/Profile.html). (B-C) Violin plots displaying the proportion of pMIR319C::GUS domain relative to leaf length in Col-0 and in tcp2;3;4;10 leaf primordia of length 50–350 μm. GUS-staining was performed either for 8 hours or for 20 hours, as indicated on the X-axis labels. The proportion of GUS domain determined using 75% (B) or 90% (C) intensity cut off. N = 15–29 leaves. Differences among samples are indicated by p-values on top of the comparisons, as determined by Mann-Whitney test. (TIF) [file pgen.1010978.s001.tif]

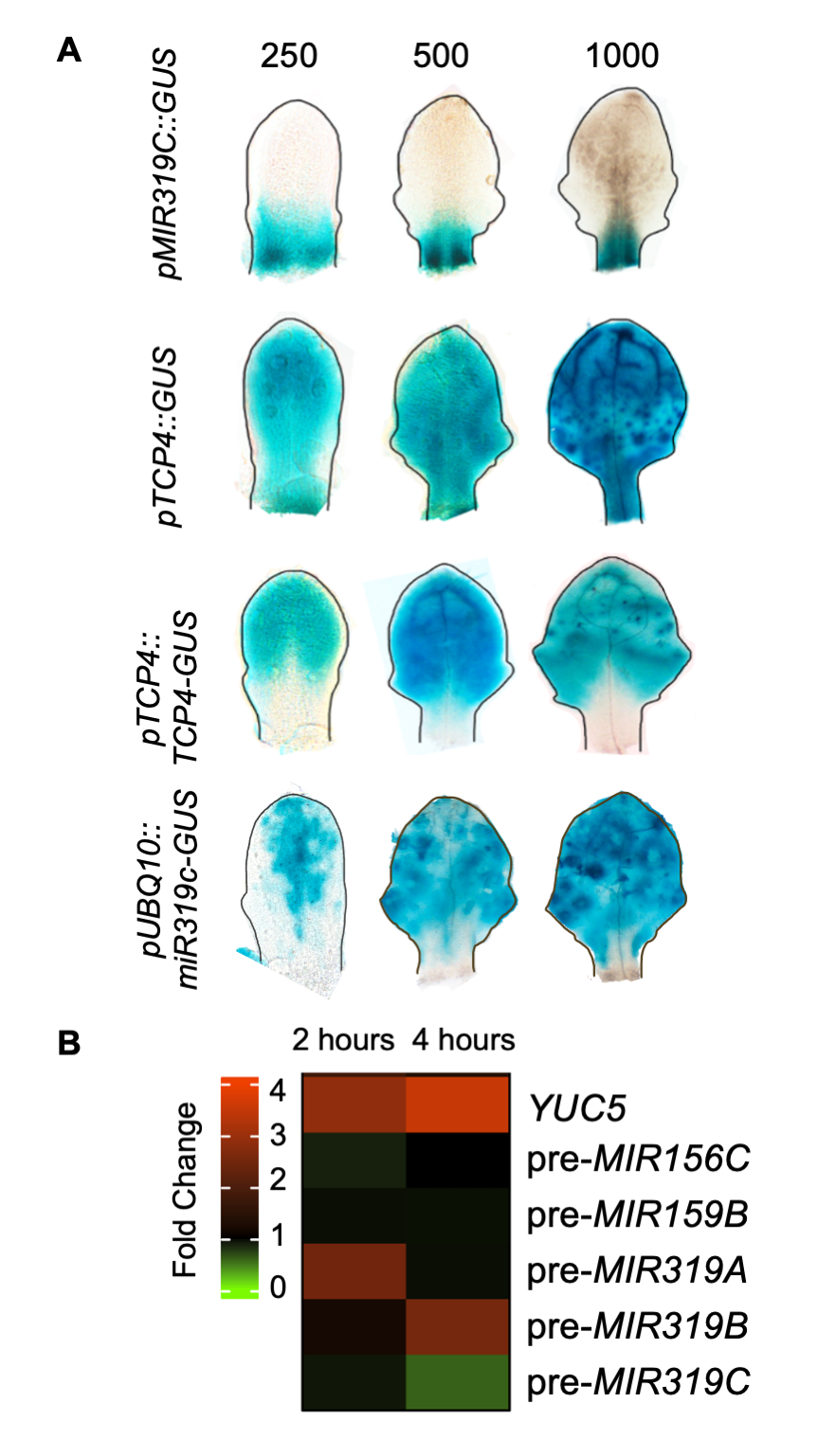

Supplement: S2 Fig — (A) Bright field images of 250, 500 and 1000 μm long wild-type leaf primordia expressing reporter constructs for MIR319C promoter (pMIR319C::GUS), TCP4 promoter (pTCP4::GUS), TCP4 protein (pTCP4::TCP4-GUS), and miR319 activity (pUBQ10::miR319c-GUS) respectively. (B) Heatmap displaying change in transcript levels of indicated genes in 9-day old jaw-D;pTCP4::rTCP4-GR (jaw-D;GR#1) [37] seedlings treated with 12 μM dexamethasone (DEX) for 2 or 4 hours. pre-MIR319C level is specifically downregulated upon 4 hours of DEX treatment as opposed to pre-MIR319A and pre-MIR319B (both upregulated) or pre-MIR156C and pre-MIR159B (unchanged). YUC5, a direct target of TCP4 [37], is used as a positive control. This is a reanalysis of microarray data reported in Challa et al [37]. (TIF) [file pgen.1010978.s002.tif]

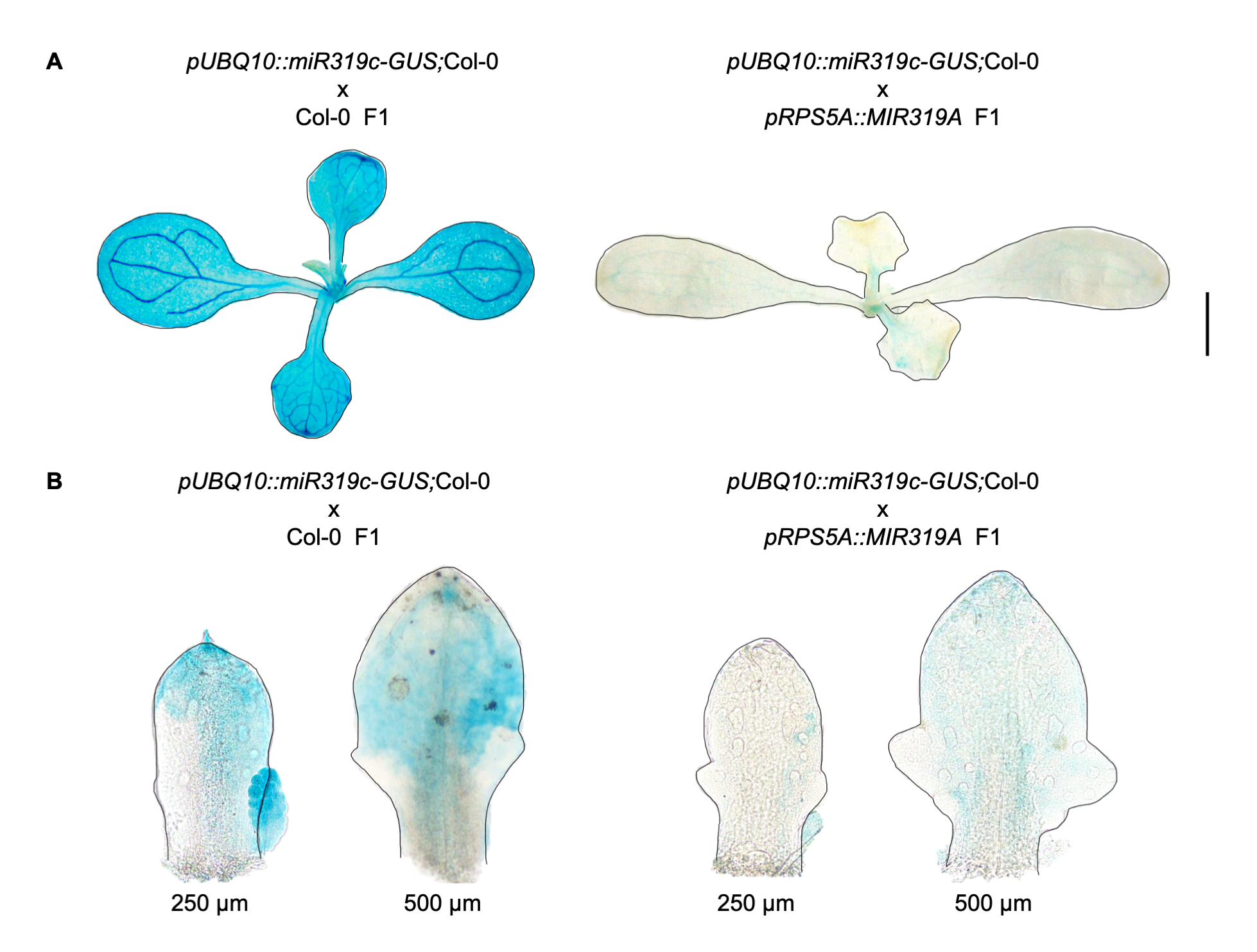

Supplement: S3 Fig — (A-B) Bright field images of (A) 9-day old whole seedlings and (B) 250 μm and 500 μm long leaf primordia at the 3rd and 4th nodes of 9-day old Col-0 and pRPS5A::MIR319A heterozygote individuals expressing pUBQ10::miR319c-GUS reporter. Scale bar in (A), 1 mm. (TIF) [file pgen.1010978.s003.tif]

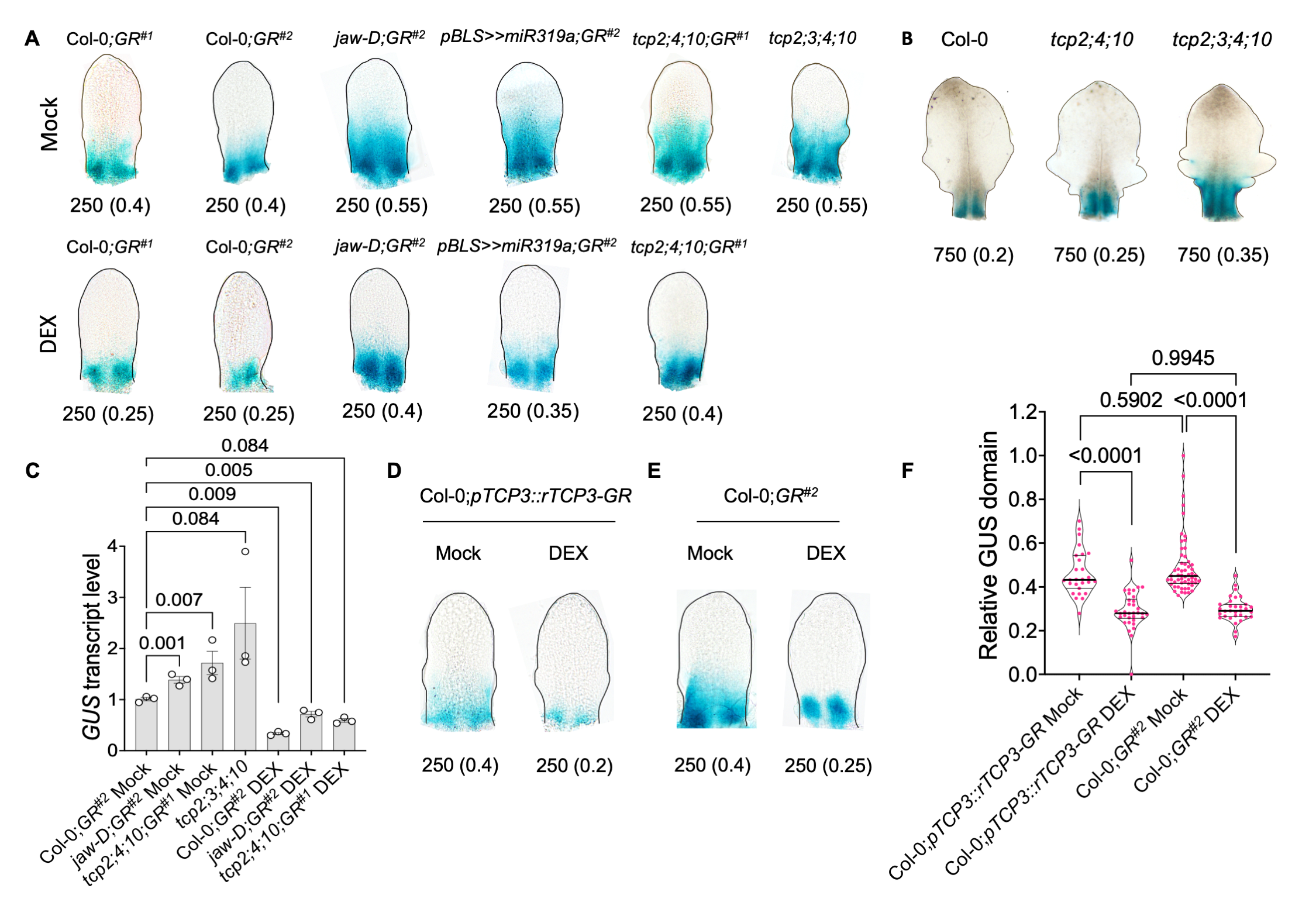

Supplement: S4 Fig — (A) Bright field images of the ethanol (Mock) or 6 μM dexamethasone (DEX)-treated 6th leaf primordia of the indicated genotypes expressing pMIR319C::GUS transgene. Numbers below the images indicate leaf length in μm and GUS domain/leaf length (in parentheses), respectively. (B) Bright field images of 750 μm long leaf primordia at the 3rd nodes of seedlings of indicated genotypes expressing pMIR319C::GUS reporter. Numbers below the images indicate leaf length in μm and GUS domain/leaf length (in parentheses), respectively. (C) GUS transcript level in the 9-day old whole seedlings of indicated genotypes treated to 0 μM (Mock) or 6 μM (DEX) dexamethasone. N = 3 biological replicates, each containing ~100 mg of tissue. Error bars indicate SEM. Differences among samples are indicated by p-values on top of the comparisons, One-way ANOVA, Dunnett’s post hoc test was performed. (D-E) Bright field images of 6th leaf primordia of the indicated genotypes expressing pMIR319C::GUS transgene. Mock and DEX in (D) and (E) indicate treatment with ethanol or 6 μM dexamethasone, respectively. The numbers below the images indicate leaf length. Proportion of GUS domain relative to leaf length is shown in the parentheses. (F) Violin plot representations of the relative pMIR319C::GUS domain (N = 27–57) in 50–250 μm long primordia on 7th-8th nodes of seedlings of indicated genotypes treated without (Mock) or with (DEX) 6 μM dexamethasone. Differences among samples are indicated by p-values on top of the plots. Two-way ANOVA, Tukey’s post hoc test was performed. (TIF) [file pgen.1010978.s004.tif]

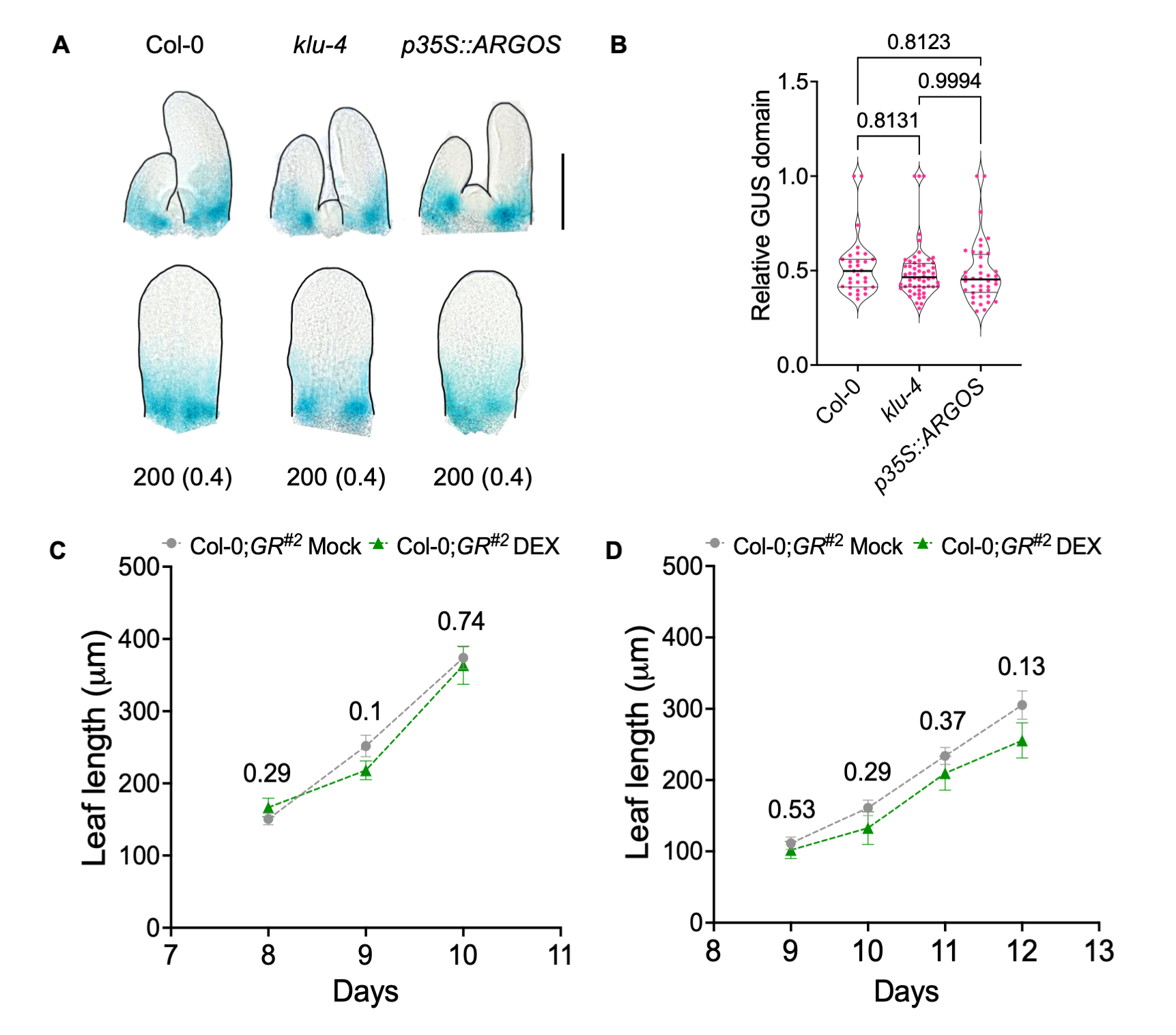

Supplement: S5 Fig — (A) Bright field images of 10-day old shoot apices and 6th leaf primordia of the indicated genotypes expressing pMIR319C::GUS transgene. Scale bar for the top panel, 100 μm. Numbers below the images refer to leaf length in μm. Proportions of GUS domain relative to leaf length are shown in the parentheses. (B) Violin plots displaying the distribution of pMIR319C::GUS domain/leaf length in leaf primordia of the genotypes indicated on the X-axis. N = 31–56 leaves. Differences among samples are indicated by p-values on top of the comparisons, One-way ANOVA, Dunnett’s post hoc test was performed. (C) and (D) Scatter plots showing mean lengths of the ethanol-treated (Mock) or 6 μM dexamethasone (DEX)-treated Col-0;GR#2 leaf primordia on corresponding days after stratification. Each grey circle or green triangle represents the mean lengths of 6th leaf primordia on 8–10 days (C) and 8th leaf primordia on 9–12 days (D) after stratification (indicated on X-axis). N = 10–35 (C) and 8–25 (D) leaves. Error bars indicate SEM. Numbers above the error bars correspond to the p-values derived from unpaired t-test with Welch’s correction for differences in variance. (TIF) [file pgen.1010978.s005.tif]

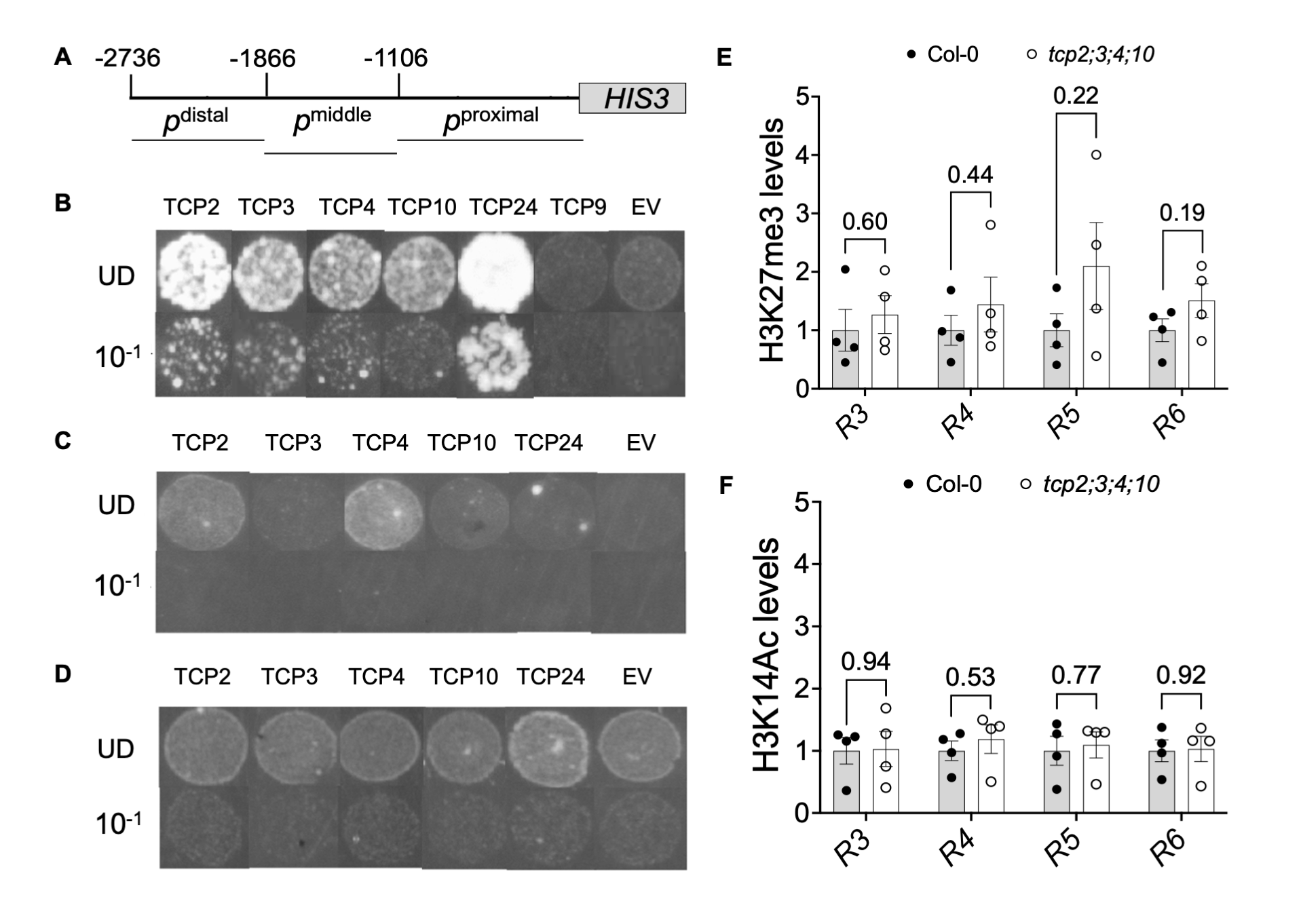

Supplement: S6 Fig — (A) Schematic representation of the HISTIDINE3 (HIS3)-based reporter construct containing 2736 bp MIR319C URR used for yeast bait generation. Grey box indicates HIS3 CDS sequence. The three horizontal lines below the URR scheme represent the truncated promoter regions, i.e., pdistal (-2736 to -1866), pmiddle (-1866 to -1106) and pproximal (-1106) used to generate the bait constructs for the yeast one-hybrid assay. (B-D) Images of yeasts harboring three truncated MIR319C promoter regions, pdistal (B), pmiddle (C) or pproximal (D) upstream to the HIS3 reporter gene and corresponding to preys as indicated, grown in the presence of inhibitory concentrations (15–20 μM) of the HIS3 inhibitor 3-amino-1,2,4-triazole (3-AT). Yeast culture suspensions (OD600 value 0.3) were spotted on Sc-His-Trp+3-AT media undiluted (UD) and at a dilution of 10−1. EV, empty vector negative control. (E) and (F) Fold enrichment of the fragments corresponding to R3-R6 regions precipitated by anti-H3K27me3 (E) and anti-H3K14Ac (F) antibody in the ChIP experiment with fragments precipitated from chromatin preparations of 9-day old Col-0 and tcp2;3;4;10 seedlings. Fold change in H3K27me3 (E) and H3K14Ac (F) levels represented as % Input and normalized to values from respective Col-0 controls. Error bars represent SEM of four biological replicates (performed in technical duplicates). Pairwise comparisons were performed using unpaired t-test. p-values are shown above the data bars. (TIF) [file pgen.1010978.s006.tif]

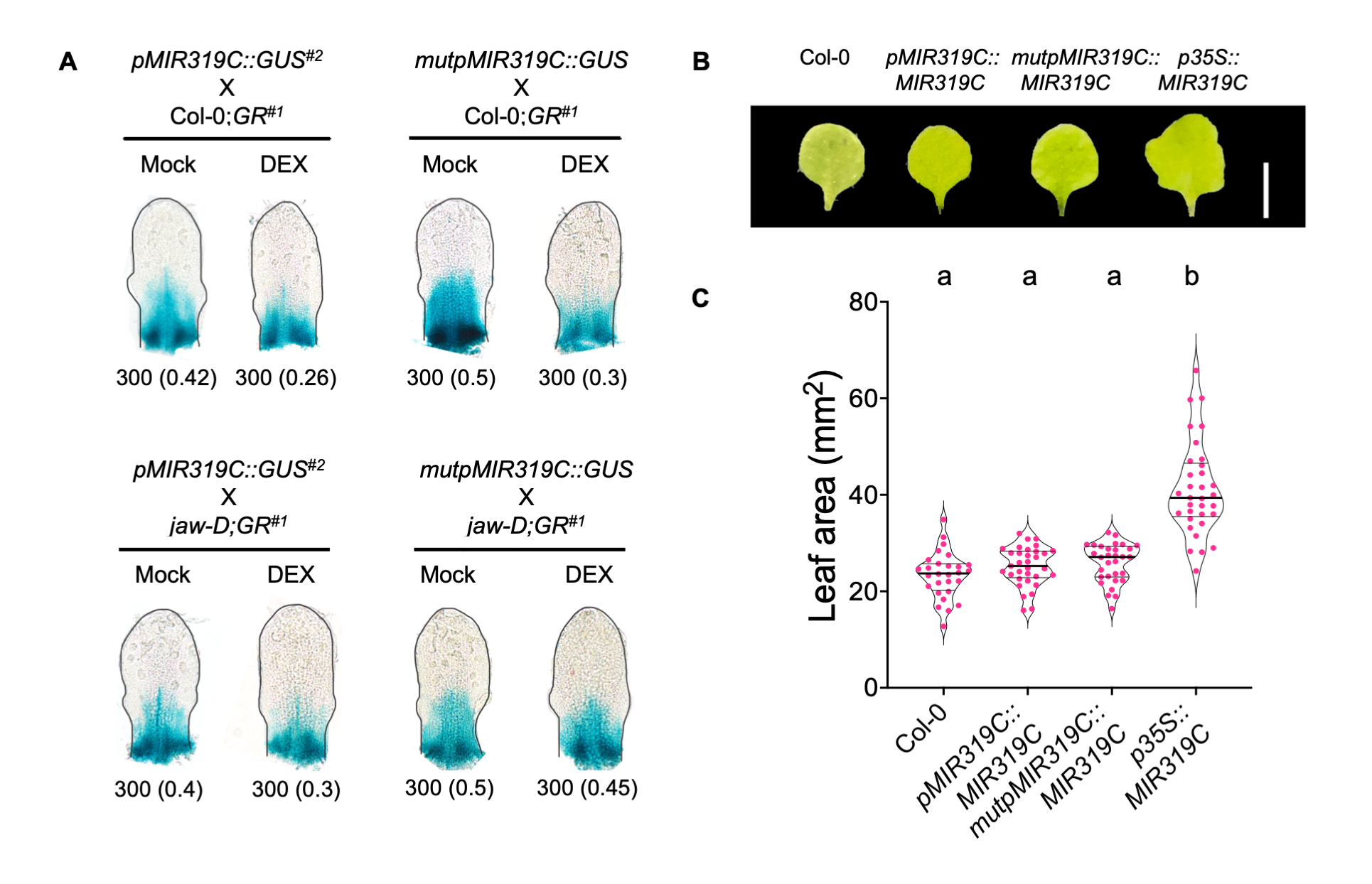

Supplement: S7 Fig — (A) Bright field images of the 5th leaf primordia from Col-0;GR#1 (top panel) and jaw-D;GR#1 (bottom panel) seedlings expressing pMIR319C::GUS#2 or mutpMIR319C::GUS transgenes as indicated. Numbers below the leaf primordia images indicate leaf length and GUS domain/leaf length (in parentheses), respectively. (B) Representative images of the 1st leaf from 25-day old seedlings of the indicated genotypes. Scale bar, 5 mm. (C) Area of the 1st leaf of the genotypes indicated on the X-axis. N = 28–33 leaves. Differences among samples are indicated by different alphabets on top of the violin plots, p<0.001. One-way ANOVA, Tukey’s post hoc test was conducted. (TIF) [file pgen.1010978.s007.tif]
