# Supplementary material for: A double-negative feedback loop between miR319c and JAW-TCPs establishes growth pattern in incipient leaf primordia in Arabidopsis thaliana
Source: PLoS Genet. 2023 Sep 28;19(9):e1010978. doi: 10.1371/journal.pgen.1010978 (PMC10564139; doi:10.1371/journal.pgen.1010978)
Supplement: Attachment — Submitted filename: Response to reviewers.docx [file pgen.1010978.s011.docx]

***Point-by-point response to the Review comments:***

**Response to editor’s comments:**

*Comment (1) both reviewers and myself were impressed with the detail of your work. reviewer 1 suggested a luciferase experiment that you can consider or explain why you don't think it is necessary. Reviewer 1 also thought a control was on another gel? I can't see what they are talking about, but please respond. I don't think you need to look at petals. Reviewer 2 has a number of grammatical and written changes for you to make. Please make these changes.*

**Our response:** We thank the editor for appreciating our work. We have explained why we think a luciferase experiment to support TCP4 binding on cis elements on *MIR319C* may not be necessary in the “response to reviewer #1 comments” section. Yes, we agree with reviewer #1 that the controls for TCP4 binding on *MIR319C* BS2 in Fig 4B could have been tested on the same gel. We have included the image of an independent EMSA experiment in the “response to reviewer #1 comments” section to support the results in Fig 4B. We have incorporated all the changes suggested by reviewer #2 and listed them in the “response to reviewer #2 comments” section of this document.

*Comment (2) Line 54. Rod-like primordium? In maize the primordium wraps the meristem and I believe it is at least a third of the perimeter in most angiosperms. I would not say "rod". A rod shape is found in leaves that have lost their abaxial/adaxial polarity.*

**Our response:** We agree with the editor that use of the term “rod-like” to describe structure of primordia is incorrect. We have now deleted the term “rod-like” from the revised MS (**line 54**).

*Comment (3) Line 82. What do you mean by several microRNA genes are intergenic? Between genes? Where else would they be?*

**Our response:** Although most plant microRNA-encoding genes (*MIRNA*) are located at the intergenic regions between protein-coding genes (*Annu. Rev. Plant Biol. 2019, 70: 489-525*), a few of them occupy the intronic/ exonic regions of protein coding genes (*Genes and Dev. 2006, 20: 3407-3425*), while a few others are located within transposable elements (*RNA. 2008, 14: 814-821*). Unlike the intergenic *MIRNA* which have their own transcriptional regulatory units, the intragenic *MIRNAs* share the same promoters as their host genes and, upon co-transcription, modulate the host transcript level (*Genes and Dev. 2006, 20: 3407-3425*; *Mol. Cell 2012, 48: 521-531*). We have now made a small modification in the revised MS as “Most plant microRNA-encoding genes (*MIRNA*) are located at intergenic regions between protein-coding genes and carry independent *cis*-elements for transcription factor (TF)-mediated regulation” to clarify this point (**lines 82-83**).

*Comment (4) Figure 2. loss of function of jaw TCPs, but it is a GR construct with dex or mock. You refer the reader to the methods, but that didn’t help.*

**Our response:** This is true, we should have explained these lines at the first citation itself. We have now incorporated this explanation in our revised MS (**lines 186-189)**.

*Comment (5) line 28. You state that mutating the cis elements produces gain of function phenotypes in the abstract, but then reading lines 348-351, one realizes it is an embryonic phenotype only. Thus, you have demonstrated amazing gene regulation, but the output phenotype is actually minor and fleeting. Maybe you should say "gain of function phenotypes in embryonic leaves" in the abstract.*

**Our response:** We agree with this, the phenotypic change is only in the embryonic leaf. We have made this correction in the abstract of our revised MS (**line 29**).

**Response to reviewer #1 comments:**

*Reviewer #1: The manuscript titled with “A double-negative feedback loop between miR319c and JAW-TCPs establishes growth pattern in incipient leaf primordia in Arabidopsis thaliana” by Shankar et al. demonstrates JAW-TCPs and miR319c mutually repress each other and generate growth polarity in early leaf primordia. The authors showed that MIR319C promoter activity was detected throughout incipient leaf primordia, and MIR319C promoter activity is abruptly downregulated in the distal domain of ~100 μm long leaf primordia with concomitant appearance of TCP4 protein in the same domain. The authors showed that MIR319C promoter activity was extended towards the distal region in tcp loss-of-function mutants, while was repressed by inducing rTCP4. These results showed that JAW-TCP activity is required for restricting MIR319C promoter activity to the proximal region. ChIP-seq, EMSA and yeast one-hybrid assay showed that TCP4 protein bound to MIR319C promoter elements. The authors further examined the relative level of histone H3K27me3 modification on MIR319C locus, which was higher at two regions of MIR319C compared to the control. They showed that TCP4 enhanced the repressive state of MIR319C chromatin. Disruption of JAW-TCP-binding sites on MIR319C promoter leads to miR319-overexpression-like phenotype.*

*The manuscript proposed a model for growth patterning in leaf primordia wherein MIR319C and JAW-TCPs repress each other and divide the uniformly growing primordia into distal differentiation zone and proximal proliferation domain. The manuscript is well-written and most of the provided data are solid. However, I have some concerns or suggestions listed below.*

*Comment (1) Transient dual-luciferase reporter assays is necessary for supporting that JAW-TCPs proximally restrict MIR319C expression domain by binding to the cis-elements in its upstream regulatory region. That is, pMIR319C-LUC or mutpMIR319C-LUC could be co-transformed with 35Spro-rTCP4 to test the binding and the importance of the cis-element in the promoter of MIR319C.*

**Our response:** We thank the Reviewer for appreciating our work. Our results from the EMSA experiment, yeast one-hybrid assay, and ChIP experiment provided biochemical evidence that the TCP4 protein binds to the upstream *cis*-element of the *MIR319C* locus and is recruited to the locus *in planta*. A successful execution of the suggested *pMIR319C-LUC* experiment would have given yet another *in vitro* support to our conclusion. Instead, we performed experiments to test the significance of the *cis* element-TCP4 interaction by expressing GUS reporter/ miR319 under the wildtype and mutated MIR319C promoter in transgenic lines. This provided us with direct and *in planta* evidence that these *cis* elements are indeed important for TCP4 interaction. Therefore, we thought that the proposed luciferase experiment would be redundant.

*Comment (2) Figure 4B, the experimental and control lanes of BS2 appear to be tested in different gels. The controls should be tested in the same gel.*

**Our response:** While preparing the original MS, we were aware that the experimental and the control lanes of BS2 are in different gels, at least in the autoradiograph presented in the MS. However, our conclusion was based on several replicates of the EMSA experiment, and the samples and the controls were in the same gel in some of the replicates. One such gel is shown below, which would perhaps convince the Reviewer about our conclusion. We can include this autoradiogram in our MS instead if the Reviewer/ Editor so desires, but we thought that would not be necessary.

*Comment (3) TCP4 is also expressed distally in the petals (Plant Comm. 2022, 3, 100309). Does the mutual repression between JAW-TCPs and MIR319 also exist in the other lateral organs such as petals? How about the expression pattern of MIR319 in the petals? The authors could discuss it in the Discussion to emphasize the significance of the repression model between JAW-TCPs and MIR319 in lateral organ formation.*

**Our response:** We thank the Reviewer for this intriguing question. We have not tested the existence of miR319-JAW-TCP DNFBL in lateral organs other than leaf. Nag et al., 2009 demonstrated that *MIR319A* and *MIR319C* are expressed throughout the early petal primordia (stage 5-7 flowers) and *MIR319A* is expressed only at the base of petals in stage 10 flowers (*PNAS. 2009, 106: 22534-22539*) whereas TCP4 protein accumulates distally in petals at the similar stage (*Plant Comm. 2022, 3: 100309*). It will be interesting to see if miR319 and JAW-TCPs mutually repress and generate their expression domains in petals. As suggested by the Reviewer, we have now discussed this possibility, and cited *Plant Comm. 2022, 3:* 100309 in our revised MS (**lines 471-475**, **994-996**).

**Response to reviewer #2 comments:**

*Reviewer #2: This paper is very nicely done. The growth analyses are excellent. The DNFBL is well demonstrated. The evidence for JAW-TCP repression via PRC2 is exciting, but I would consider it still weak based on what’s presented in the paper. I detail my thoughts on this and other minor issue below:*

*Comment* (*1) Line 107 – change “contrary” to “conversely”*

**Our response:** We have incorporated this change (**line 108**).

*Comment (2) Line 109 – change “suggesting” to “demonstrating”*

**Our response:** True, it should be ‘demonstrating’. We have made the change (**line 110**).

*Comment (3) Line 120 – change “proposition” to “mechanism”*

*Comment (4) Lines 120-122 – I can think of “simpler” models than a DNFBL. See additional comment below. Just say something like, “A simple and robust mechanism for dividing an incipient primordium…”.*

**Our response:** This is a very nice suggestion. We have now changed it to “A simple and robust mechanism for…” in our revised MS (**lines 120-121**).

*Comment (5) Line 131 – change “complementary” to “compared”*

**Our response:** The suggested change incorporated (**line 132**).

*Comment (6) Line 155 – “detected till leaves”*

**Our response:** We have made this change (**line 156**).

*Comment (7) Line 182 – change to “with the concomitant”*

**Our response:** We have incorporated this change in our revised MS (now **line 183**).

*Comment (8) Figure 2B – remove 1.2 tick from y-axis.*

**Our response:** We have removed 1.2 tick from the Y-axis in **Fig 2B** in our revised MS.

*Comment (9) Line 253 – change “activity at the base” to “activity to the base”*

**Our response:** We have incorporated this change (**line 257**).

*Comment (10) Lines 328-329 – I don’t understand the logic for why endogenous TCP copies interfere with an exogenous copies ability to bind the MIR319 promoter. The experimental manipulation is the mutations in the promoter of MIR319C, which are unchanged whether endogenous copies exist or not. Those same endogenous copies didn’t prevent you from detecting a difference in GUS when you induce TCP4.*

**Our response:** I think we could not put forth our arguments clearly in this case, and I will try to clarify here. We observed that, in *jaw-D* leaves with very low level of *JAW-TCPs* (10-30 fold lower; *Nature 2003; 425: 257–263*), induction of rTCP4 reduced *pMIR319C::GUS* domain proximally, but failed to do so when the *cis*-element was mutated in the *mutpMIR319C::GUS* transgene, suggesting that TCP4 requires the wildtype *cis* element to repress *MIR319C* in the *jaw-D* background. However, rTCP4 induction restricted the *pMIR319C* domain more proximally irrespective of the mutation in the *cis* element, in the Col-0 background where all the JAW-TCPs are present at the endogenous level. It is possible that these JAW-TCP proteins help the exogenous rTCP4 bind to even the mutated *cis* elements, possibly by forming hetero dimers. Formation of heterodimers among the JAW-TCP members have been demonstrated earlier (*Plant Cell 2010; 22: 1174–1189*; *J. Exp. Bot. 2013; 64: 5673–5685*). We have now incorporated this clarification in our revised MS (**lines 332-334 in *Results*, and in lines 438-444 in *Discussion***).

*Comment (11) Lines 351-353 – I don’t think the current evidence supports this statement.*

**Our response:** We agree that the evidence for this conclusion is not very strong. We attempted to address how our result fits into overall leaf form by mutating the TCP4-binding elements of *MIR319C*. Whereas we observed *MIR319C* gain-of-function effect in the embryonic leaf form when TCP4-binding was compromised, the genetic intervention did not alter rosette leaf form, perhaps because of the presence of a large number of JAW-TCPs in the Col-0 background. Though functional redundancy among *JAW-TCP*s makes it difficult to carry our genetic studies, our work provided some proof-of-principle evidence that the JAW-TCP proteins repress *MIR319C* transcription in planta, the absence of which can alter cotyledon form. Based on this, we have now made minor changes in our statement to make our claim less strong (**lines 357-358**).

*Comment (12) Lines 361-363 – I’m not sure I agree with this statement. It seems that proximal expression (and acropetal diffusion) of MIR319C would be sufficient to achieve a polar pattern between these two factors. A DNFBL has other benefits (and seems to be what has evolved), but I’d hardly call it “minimalistic”.*

**Our response:** We agree that DNFBL may not be the only option. We have now made the suggested changes (**lines 367-368)**.

*Comment (13) Lines 433-436 – I don’t understand this statement. TCP4 didn’t require other JAW-TCPs for MIR319C repression when the wildtype MIR319C promoter was used.*

**Our response:** We have addressed this point above in our response to *Comment 10*. We have now modified the statement as per the suggestion of the Reviewer (**lines 438-444**).

*Comment (14) Line 1045 – change “alphabets” to “letters”*

**Our response:** We have incorporated this change (**line 1023**).

*Comment (15) Figure 1 and 2 – I don’t think I found it discussed anywhere in the paper, but it is interesting that pMIR319::GUS shows a high level of distal exclusion even in TCP mutants, despite initially having a uniform pattern of expression.*

**Our response:** This is indeed an important point, and we had discussed it in our original MS. We have now extended it a bit more and highlighted it in our revised MS (**lines 396-403**). In short, the possible candidates other than *JAW-TCP*s that repress *MIR319C* at the distal region may include other class II *TCP* homologues, and members belonging to the *NGATHA* and *KNOX-II* gene families.

**List of changes in the revised MS:**

(1) Line 29- Inserted “in the embryonic leaves”.

(2) Line 54- deleted “a simple rod-like primordium”, inserted “initiation”

(3) Line 82- Replaced “Several” with “Most”

(4) Lines 82-83- changed “intergenic” to “located at intergenic regions between protein coding genes”

(4) Line 108- Replaced “On the contrary” with “Conversely”

(5) Line 110- Changed “suggesting” to “demonstrating”

(6) Lines 120-121- Replaced “The most parsimonious proposition of ” with “A simple and robust mechanism for”

(7) Line 132- Changed “complementary” to “compared”

(8) Line 156- Changed “detected till leaves”

(9) Line 183- Added “the” preposition

(10) Lines 186-189- replaced “Col-0;*GR^#1^* and Col-0;*GR^#2^*; see Materials for details” to “In plants with endogenous *JAW-TCP* function (mock-treated Col-0;*GR^#1^* and Col-0;*GR^#2^*; though these lines contain a dexamethasone-inducible miR319-resistant TCP4, they resemble wildtype under non-inductive condition as reported earlier by Challa et al., 2016; also see **S1 Table for details**)”

(11) Line 192- Inserted “mock-treated”

(12) Lines 193- Inserted “mock-treated”

(13) Line 194- Inserted “see S1 Table for details”

(14) Figure 2B- Removed 1.2 tick on y-axis

(15) Line 257- Replaced “at” with “to”

(16) Lines 333- Changed “level of JAW-TCP proteins” to JAW-TCPs

(17) Lines 357-358- Inserted “ an effect that is revealed only when the level of endogenous JAW-TCPs is reduced”

(18) Lines 367-368- Replaced “would be a minimalistic proposal” with “could be one of the possible ways”

(19) Lines 399-400- Inserted “including other class II *TCP* homologues”

(20) Lines 438-444 Deleted “Interestingly, TCP4 failed to downregulate the activity of *MIR319C* promoter with mutated *cis*-elements only in the *jaw-D* leaf primordia but not in wild-type, suggesting that TCP4 requires other JAW-TCPs for *MIR319C* repression. However, the lack of JAW-TCP binding did not further extend the *MIR319C* expression domain both in the Col-0 and *jaw-D* backgrounds (**Figs. 5B and 5C**), perhaps indicating the existence of an indirect pathway for JAW-TCP-mediated *MIR319C* repression”. Inserted “We observed that dominant TCP4 failed to downregulate the activity of *MIR319C* promoter with mutated *cis*-elements only in the *jaw-D* leaf primordia but not in wild-type (**Figs. 5B and 5C**). It is possible that the endogenous JAW-TCP proteins in Col-0 help the exogenous rTCP4 bind even to the mutated *cis* element, possibly by forming hetero dimers. Formation of heterodimers among the JAW-TCP members had been demonstrated earlier (Aggarwal et al., 2010; Danisman et al., 2013)”.

(21) Lines 471-475- Inserted “Both *MIR319A* and *MIR319C* are expressed throughout the earliest stages of petal primordia (Nag et al., 2009). Interestingly, *MIR319A* is only active at the base of the petals at a stage when TCP4 strongly accumulates only at the distal region (Zheng et al., 2022). It will be interesting to investigate if miR319-JAW-TCP DNFBL serves as a general mechanism to regulate spatiotemporal growth of lateral organs”

(22) Line 994-996- Inserted new citation-Zheng et al., 2022.

(23) Line 1023- Changed “alphabets” to “letters”
