# Supplementary material for: A double-negative feedback loop between miR319c and JAW-TCPs establishes growth pattern in incipient leaf primordia in Arabidopsis thaliana
Source: PLoS Genet. 2023 Sep 28;19(9):e1010978. doi: 10.1371/journal.pgen.1010978 (PMC10564139; doi:10.1371/journal.pgen.1010978)
Supplement: Peer review history

# Peer review: Decision Letter 0

**DOI:** 10.1371/journal.pgen.1010978.r001

**Authors:** Sarah Hake, Claudia Köhler

30 Aug 2023

Dear Dr Nath,

Thank you very much for submitting your Research Article entitled 'A double-negative feedback loop between miR319c and JAW-TCPs establishes growth pattern in incipient leaf primordia in Arabidopsis thaliana' to PLOS Genetics.

The manuscript was fully evaluated at the editorial level and by independent peer reviewers. The reviewers appreciated the attention to an important topic but identified some concerns that we ask you address in a revised manuscript.

We therefore ask you to modify the manuscript according to the review recommendations. Your revisions should address the specific points made by each reviewer.

In addition we ask that you:

1) Provide a detailed list of your responses to the review comments and a description of the changes you have made in the manuscript.

2) Upload a Striking Image with a corresponding caption to accompany your manuscript if one is available (either a new image or an existing one from within your manuscript). If this image is judged to be suitable, it may be featured on our website. Images should ideally be high resolution, eye-catching, single panel square images. For examples, please browse our archive. If your image is from someone other than yourself, please ensure that the artist has read and agreed to the terms and conditions of the Creative Commons Attribution License. Note: we cannot publish copyrighted images.

We hope to receive your revised manuscript within the next 30 days. If you anticipate any delay in its return, we would ask you to let us know the expected resubmission date by email to plosgenetics@plos.org.

If present, accompanying reviewer attachments should be included with this email; please notify the journal office if any appear to be missing. They will also be available for download from the link below. You can use this link to log into the system when you are ready to submit a revised version, having first consulted our Submission Checklist.

While revising your submission, please upload your figure files to the Preflight Analysis and Conversion Engine (PACE) digital diagnostic tool. PACE helps ensure that figures meet PLOS requirements. To use PACE, you must first register as a user. Then, login and navigate to the UPLOAD tab, where you will find detailed instructions on how to use the tool. If you encounter any issues or have any questions when using PACE, please email us at figures@plos.org.

Please be aware that our data availability policy requires that all numerical data underlying graphs or summary statistics are included with the submission, and you will need to provide this upon resubmission if not already present. In addition, we do not permit the inclusion of phrases such as "data not shown" or "unpublished results" in manuscripts. All points should be backed up by data provided with the submission.

To enhance the reproducibility of your results, we recommend that you deposit your laboratory protocols in protocols.io, where a protocol can be assigned its own identifier (DOI) such that it can be cited independently in the future. Additionally, PLOS ONE offers an option to publish peer-reviewed clinical study protocols. Read more information on sharing protocols at https://plos.org/protocols?utm_medium=editorial-email&utm_source=authorletters&utm_campaign=protocols

Please review your reference list to ensure that it is complete and correct. If you have cited papers that have been retracted, please include the rationale for doing so in the manuscript text, or remove these references and replace them with relevant current references. Any changes to the reference list should be mentioned in the rebuttal letter that accompanies your revised manuscript. If you need to cite a retracted article, indicate the article’s retracted status in the References list and also include a citation and full reference for the retraction notice.

PLOS has incorporated Similarity Check, powered by iThenticate, into its journal-wide submission system in order to screen submitted content for originality before publication. Each PLOS journal undertakes screening on a proportion of submitted articles. You will be contacted if needed following the screening process.

To resubmit, you will need to go to the link below and 'Revise Submission' in the 'Submissions Needing Revision' folder.

Please let us know if you have any questions while making these revisions.

Yours sincerely,

Sarah Hake

Academic Editor

PLOS Genetics

Claudia Köhler

Section Editor

PLOS Genetics

Dear Authors

both reviewers and myself were impressed with the detail of your work. reviewer 1 suggested a luciferase experiment that you can consider or explain why you don't think it is necessary. Reviewer 1 also thought a control was on another gel? I can't see what they are talking about, but please respond. I don't think you need to look at petals. Reviewer 2 has a number of grammatical and written changes for you to make. Please make these changes.

I had a couple comments.

Line 54. Rod-like primordium? In maize the primordium wraps the meristem and I believe it is at least a third of the perimeter in most angiosperms. I would not say "rod". A rod shape is found in leaves that have lost their abaxial/adaxial polarity.

Line 82. What do you mean by several microRNA genes are intergenic? Between genes? Where else would they be?

Figure 2. loss of function of jaw TCPs, but it is a GR construct with dex or mock. You refer the reader to the methods, but that didn’t help.

line 28. You state that mutating the cis elements produces gain of function phenotypes in the abstract, but then reading lines 348-351, one realizes it is an embryonic phenotype only. Thus, you have demonstrated amazing gene regulation, but the output phenotype is actually minor and fleeting. Maybe you should say "gain of function phenotypes in embryonic leaves" in the abstract.

Reviewer's Responses to Questions

**Comments to the Authors:**

**Please note here if the review is uploaded as an attachment.**

Reviewer #1: The manuscript titled with “A double-negative feedback loop between miR319c and JAW-TCPs establishes growth pattern in incipient leaf primordia in Arabidopsis thaliana” by Shankar et al. demonstrates JAW-TCPs and miR319c mutually repress each other and generate growth polarity in early leaf primordia. The authors showed that MIR319C promoter activity was detected throughout incipient leaf primordia, and MIR319C promoter activity is abruptly downregulated in the distal domain of ~100 μm long leaf primordia with concomitant appearance of TCP4 protein in the same domain. The authors showed that MIR319C promoter activity was extended towards the distal region in tcp loss-of-function mutants, while was repressed by inducing rTCP4. These results showed that JAW-TCP activity is required for restricting MIR319C promoter activity to the proximal region. ChIP-seq, EMSA and yeast one-hybrid assay showed that TCP4 protein bound to MIR319C promoter elements. The authors further examined the relative level of histone H3K27me3 modification on MIR319C locus, which was higher at two regions of MIR319C compared to the control. They showed that TCP4 enhanced the repressive state of MIR319C chromatin. Disruption of JAW-TCP-binding sites on MIR319C promoter leads to miR319-overexpression-like phenotype.

The manuscript proposed a model for growth patterning in leaf primordia wherein MIR319C and JAW-TCPs repress each other and divide the uniformly growing primordia into distal differentiation zone and proximal proliferation domain. The manuscript is well-written and most of the provided data are solid. However, I have some concerns or suggestions listed below.

1. Transient dual-luciferase reporter assays is necessary for supporting that JAW-TCPs proximally restrict MIR319C expression domain by binding to the cis-elements in its upstream regulatory region. That is, pMIR319C-LUC or mutpMIR319C-LUC could be co-transformed with 35Spro-rTCP4 to test the binding and the importance of the cis-element in the promoter of MIR319C.

2. Figure 4B, the experimental and control lanes of BS2 appear to be tested in different gels. The controls should be tested in the same gel.

3. TCP4 is also expressed distally in the petals (Plant Comm. 2022, 3, 100309). Does the mutual repression between JAW-TCPs and MIR319 also exist in the other lateral organs such as petals? How about the expression pattern of MIR319 in the petals? The authors could discuss it in the Discussion to emphasize the significance of the repression model between JAW-TCPs and MIR319 in lateral organ formation.

Reviewer #2: This paper is very nicely done. The growth analyses are excellent. The DNFBL is well demonstrated. The evidence for JAW-TCP respression via PRC2 is exciting, but I would consider it still weak based on what’s presented in the paper. I detail my thoughts on this and other minor issue below:

Line 107 – change “contrary” to “conversely”

Line 109 – change “suggesting” to “demonstrating”

Line 120 – change “proposition” to “mechanism”

Lines 120-122 – I can think of “simpler” models than a DNFBL. See additional comment below. Just say something like, “A simple and robust mechanism for dividing an incipient primordium…”.

Line 131 – change “complementary” to “compared”

Line 155 – “detected till leaves”

Line 182 – change to “with the concomitant”

Figure 2B – remove 1.2 tick from y-axis.

Line 253 – change “activity at the base” to “activity to the base”

Lines 328-329 – I don’t understand the logic for why endogenous TCP copies interfere with an exogenous copies ability to bind the MIR319 promoter. The experimental manipulation is the mutations in the promoter of MIR319C, which are unchanged whether endogenous copies exist or not. Those same endogenous copies didn’t prevent you from detecting a difference in GUS when you induce TCP4.

Lines 351-353 – I don’t think the current evidence supports this statement.

Lines 361-363 – I’m not sure I agree with this statement. It seems that proximal expression (and acropetal diffusion) of MIR319C would be sufficient to achieve a polar pattern between these two factors. A DNFBL has other benefits (and seems to be what has evolved), but I’d hardly call it “minimalistic”.

Lines 433-436 – I don’t understand this statement. TCP4 didn’t require other JAW-TCPs for MIR319C repression when the wildtype MIR319C promoter was used.

Line 1045 – change “alphabets” to “letters”

Figure 1 and 2 – I don’t think I found it discussed anywhere in the paper, but it is interesting that pMIR319::GUS shows a high level of distal exclusion even in TCP mutants, despite initially having a uniform pattern of expression.

**********

**Have all data underlying the figures and results presented in the manuscript been provided?**

Large-scale datasets should be made available via a public repository as described in the *PLOS Genetics* data availability policy, and numerical data that underlies graphs or summary statistics should be provided in spreadsheet form as supporting information.

Reviewer #1: Yes

Reviewer #2: Yes

**********

PLOS authors have the option to publish the peer review history of their article (what does this mean?). If published, this will include your full peer review and any attached files.

If you choose “no”, your identity will remain anonymous but your review may still be made public.

**Do you want your identity to be public for this peer review?** For information about this choice, including consent withdrawal, please see our Privacy Policy.

Reviewer #1: **Yes: **GENJI QIN

Reviewer #2: No

# Author comment: Author response to Decision Letter 0

**DOI:** 10.1371/journal.pgen.1010978.r002

13 Sep 2023

Attachment Submitted filename: Response to reviewers.docx Click here for additional data file.

# Editor report: Decision Letter 1

**DOI:** 10.1371/journal.pgen.1010978.r003

**Authors:** Sarah Hake, Claudia Köhler

17 Sep 2023

Dear Dr Nath,

We are pleased to inform you that your manuscript entitled "A double-negative feedback loop between miR319c and JAW-TCPs establishes growth pattern in incipient leaf primordia in Arabidopsis thaliana" has been editorially accepted for publication in PLOS Genetics. Congratulations!

Before your submission can be formally accepted and sent to production you will need to complete our formatting changes, which you will receive in a follow up email. Please be aware that it may take several days for you to receive this email; during this time no action is required by you. Please note: the accept date on your published article will reflect the date of this provisional acceptance, but your manuscript will not be scheduled for publication until the required changes have been made.

Once your paper is formally accepted, an uncorrected proof of your manuscript will be published online ahead of the final version, unless you’ve already opted out via the online submission form. If, for any reason, you do not want an earlier version of your manuscript published online or are unsure if you have already indicated as such, please let the journal staff know immediately at plosgenetics@plos.org.

In the meantime, please log into Editorial Manager at https://www.editorialmanager.com/pgenetics/, click the "Update My Information" link at the top of the page, and update your user information to ensure an efficient production and billing process. Note that PLOS requires an ORCID iD for all corresponding authors. Therefore, please ensure that you have an ORCID iD and that it is validated in Editorial Manager. To do this, go to ‘Update my Information’ (in the upper left-hand corner of the main menu), and click on the Fetch/Validate link next to the ORCID field.  This will take you to the ORCID site and allow you to create a new iD or authenticate a pre-existing iD in Editorial Manager.

If you have a press-related query, or would like to know about making your underlying data available (as you will be aware, this is required for publication), please see the end of this email. If your institution or institutions have a press office, please notify them about your upcoming article at this point, to enable them to help maximise its impact. Inform journal staff as soon as possible if you are preparing a press release for your article and need a publication date.

Thank you again for supporting open-access publishing; we are looking forward to publishing your work in PLOS Genetics!

Yours sincerely,

Sarah Hake

Academic Editor

PLOS Genetics

Claudia Köhler

Section Editor

PLOS Genetics

www.plosgenetics.org

Twitter: @PLOSGenetics

----------------------------------------------------

Comments from the reviewers (if applicable):

----------------------------------------------------

**Data Deposition**

If you have submitted a Research Article or Front Matter that has associated data that are not suitable for deposition in a subject-specific public repository (such as GenBank or ArrayExpress), one way to make that data available is to deposit it in the Dryad Digital Repository. As you may recall, we ask all authors to agree to make data available; this is one way to achieve that. A full list of recommended repositories can be found on our website.

The following link will take you to the Dryad record for your article, so you won't have to re‐enter its bibliographic information, and can upload your files directly: 

http://datadryad.org/submit?journalID=pgenetics&manu=PGENETICS-D-23-00877R1

More information about depositing data in Dryad is available at http://www.datadryad.org/depositing. If you experience any difficulties in submitting your data, please contact help@datadryad.org for support.

Additionally, please be aware that our data availability policy requires that all numerical data underlying display items are included with the submission, and you will need to provide this before we can formally accept your manuscript, if not already present.

----------------------------------------------------

**Press Queries**

If you or your institution will be preparing press materials for this manuscript, or if you need to know your paper's publication date for media purposes, please inform the journal staff as soon as possible so that your submission can be scheduled accordingly. Your manuscript will remain under a strict press embargo until the publication date and time. This means an early version of your manuscript will not be published ahead of your final version. PLOS Genetics may also choose to issue a press release for your article. If there's anything the journal should know or you'd like more information, please get in touch via plosgenetics@plos.org.

# Editor report: Acceptance letter

**DOI:** 10.1371/journal.pgen.1010978.r004

**Authors:** Sarah Hake, Claudia Köhler

26 Sep 2023

PGENETICS-D-23-00877R1 

A double-negative feedback loop between miR319c and JAW-TCPs establishes growth pattern in incipient leaf primordia in Arabidopsis thaliana 

Dear Dr Nath, 

We are pleased to inform you that your manuscript entitled "A double-negative feedback loop between miR319c and JAW-TCPs establishes growth pattern in incipient leaf primordia in Arabidopsis thaliana" has been formally accepted for publication in PLOS Genetics! Your manuscript is now with our production department and you will be notified of the publication date in due course.

The corresponding author will soon be receiving a typeset proof for review, to ensure errors have not been introduced during production. Please review the PDF proof of your manuscript carefully, as this is the last chance to correct any errors. Please note that major changes, or those which affect the scientific understanding of the work, will likely cause delays to the publication date of your manuscript. 

Soon after your final files are uploaded, unless you have opted out or your manuscript is a front-matter piece, the early version of your manuscript will be published online. The date of the early version will be your article's publication date. The final article will be published to the same URL, and all versions of the paper will be accessible to readers.

Thank you again for supporting PLOS Genetics and open-access publishing. We are looking forward to publishing your work! 

With kind regards,

Zsofi Zombor

PLOS Genetics

On behalf of:

The PLOS Genetics Team

Carlyle House, Carlyle Road, Cambridge CB4 3DN | United Kingdom

plosgenetics@plos.org | +44 (0) 1223-442823

plosgenetics.org | Twitter: @PLOSGenetics
